# The effect of acute aerobic exercise intensity on indices of blood flow to metastatic liver tumours using non-invasive imaging: a preliminary study

**DOI:** 10.1007/s00421-026-06139-3

**Published:** 2026-03-11

**Authors:** Catherine Seet-Lee, Jasmine Yee, Jillian L. Clarke, Thomas Chalmers Braithwaite, Kate M. Edwards

**Affiliations:** 1 Sydney School of Health Sciences, Faculty of Medicine and Health, The University of Sydney, Sydney, Australia; 2 Charles Perkins Centre, The University of Sydney, Sydney, Australia; 3 Department of Supportive Care and Integrative Oncology, Chris O’Brien Lifehouse, Sydney, Australia; 4 School of Psychology, Faculty of Science, The University of Sydney, Sydney, Australia

**Keywords:** Exercise, Blood flow, Tumour, Ultrasound

## Abstract

**Introduction:**

Tumours have heterogenous and dysfunctional vasculature that can cause a therapeutic barrier for intravenous chemotherapy delivery. As tumours do not vasoconstrict, aerobic exercise may acutely increase tumour blood flow through increased cardiac output and potentially increase chemotherapy delivery. While pre-clinical studies have demonstrated increased tumour blood flow with moderate intensity aerobic exercise, no clinical studies have assessed this effect. This study examined the effect of acute aerobic exercise at varying intensities on tumour blood flow in people with liver metastases.

**Methods:**

Participants with stage IV cancer with liver metastases performed an exercise test followed by three 5-minute bouts of light, moderate and high intensity cycling. Doppler ultrasound assessed blood flow to the hepatic artery (control) and liver tumour at baseline and after each exercise bout for 10 min.

**Result:**

Eight participants completed the study, however, three were excluded from analysis due to a lack of sonographer confidence to identify and measure tumour vessels, due to small size and poor accessibility. There was 152% increase in blood flow (peak systolic velocity) to the tumour after moderate intensity exercise (24.63 ± 5.66 to 37.56 ± 5.91, *p* = 0.043), and an increase in tumour and hepatic arterial resistance (resistive index) after high intensity exercise (0.65 ± 0.14 to 0.74 ± 0.15 and 0.81 ± 0.15 respectively, *p* = 0.043).

**Conclusion:**

Moderate intensity exercise acutely increases blood flow to metastatic liver tumours. These findings support future work to examine whether aerobic exercise improves clinical outcomes such as chemotherapy delivery and efficacy, particularly when delivered concurrent to infusion.

**Supplementary Information:**

The online version contains supplementary material available at 10.1007/s00421-026-06139-3.

## Introduction

Intravenous chemotherapy is a common anti-cancer treatment administered to destroy cancer cells in tumours. Efficient chemotherapy relies on adequate doses of the chemotherapy agent to be delivered to the tumour, specifically the tumour centre, via the vascular system (Jain 2013). Compared to healthy tissue, the tumour microenvironment has an abnormal structure and function (Wiggins et al. 2018). The tumour vascular network has a heterogenous vessel distribution leading to areas of both high and low blood vessel density. The vessels themselves are immature, lacking a normal vasoconstriction response (McCullough et al. 2014; Siemann 2015), and have irregular endothelial cell distribution causing high permeability and increased interstitial pressure (Jain 1990). In the tumour centre, these abnormalities result in collapsed vessels and reduced blood flow (Wu et al. 2013) leading to areas of low perfusion and hypoxic regions (Schumacher et al. 2021; Wiggins et al. 2018). This can cause a therapeutic barrier for chemotherapy delivery leading to less efficient chemotherapy response and poorer treatment outcomes.

Aerobic exercise is a promising intervention that may improve intravenous chemotherapy delivery to tumours and potentially modulate the dysfunctional tumour microenvironment (Wiggins et al. 2018). Aerobic exercise acutely increases cardiac output and blood flow to tissues, and is mediated by vasoconstriction (shunting blood away from inactive tissue) and vasodilation (increasing vessel dilation to increase blood flow volume into active tissue) (Brooks et al. 2005). In tumours, due to the abnormal vessel structure and lack of vasoconstriction response, an increase in cardiac output with aerobic exercise may result in increased blood flow regardless of tissue activity signaling (Wiggins et al. 2018). With increased tumour blood flow, there is the possibility that aerobic exercise could increase intravenous chemotherapy delivery directly to the tumour.

Pre-clinical studies have demonstrated that acute moderate intensity aerobic exercise can increase tumour blood flow and induce favourable changes in tumour vasculature that promote tumour perfusion (Gomes-Santos et al. 2024; McCullough et al. 2013). However, there is currently no clinical evidence for the immediate acute effects of aerobic exercise on tumour blood flow in humans.

This study aims to address this gap and examine the effect of acute aerobic exercise at varying intensities on tumour blood flow in people with liver metastases, using non-invasive measurement techniques.

## Methods

### Participants

Participants were recruited from Chris O’Brien Lifehouse, Sydney, Australia (ANZCTR Trial ID ACTRN12619000846123). Participants were eligible if they met the following criteria: aged >18 years, diagnosed with stage IV cancer of any primary cancer, Eastern Cooperative Oncology Group (ECOG) 0–2 (Oken et al. 1982) and had at least 1 liver metastasis ≥1 cm. Participants were excluded if they presented with an acute systemic infection or fever, had any condition that is a contraindication to exercise or were currently taking beta-blocker medication. Participants who were eligible were referred by their treating oncologist.

### Study design

The study took place at the University of Sydney, Sydney, Australia (ethics approval X19-0123 & 2019/ETH00544). After providing informed consent and completing demographic questionnaires, participants had their most accessible liver tumour identified by real-time ultrasound, and both this tumour vessel and their healthy hepatic artery (as a comparator) measured with Doppler, using a curved 1 - 5 MHz transducer on a Sparq ultrasound machine (Philips Healthcare, Andover, Massachusetts, USA). Doppler ultrasound was chosen to measure blood flow as it is a non-invasive validated measurement of blood flow (Dodd et al. 1994). Participants then undertook a YMCA submaximal cycle ergometer test on a recumbent bike (Lode Corival Recumbent) with expired gas analysis using a metabolic cart (Medgraphics Ultima CardiO2). After 10 minutes recovery, participants had ultrasound measures of their liver tumour vessel and hepatic artery.

**Fig. 1:**
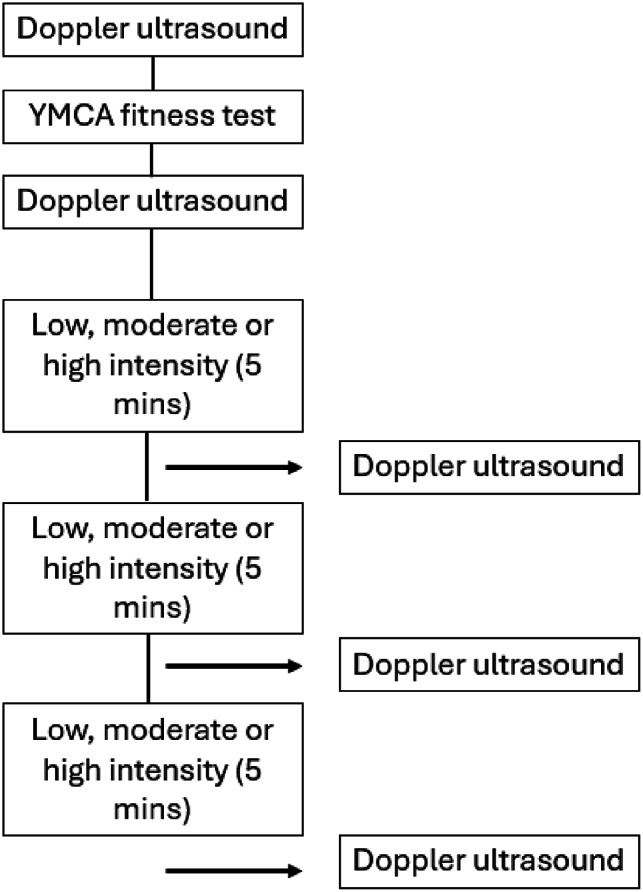
Study schema

Data from the YMCA test was used to calculate predicted VO_2peak_. Participants with predicted VO_2peak_ ≤18 ml/kg/min performed a single 5-minute bout of moderate intensity cycling followed by 10 minutes of ultrasound measures. Participants with VO_2 peak_ > 18 ml/kg/min performed three 5-minute bouts of light (27–37% VO_2max_/20–30% heart rate reserve), moderate (46–56% VO_2max_/40–50% heart rate reserve) and high intensity (64–74% VO_2max_/60–70% heart rate reserve) cycling in a randomised order. This cut off for multiple intensity bout eligibility was based on a previous examination of fitness in individuals with liver metastases, which reported an average aerobic fitness (VO_2peak_) of 18 ml/kg/min and demonstrated that this population were able to complete a high intensity exercise test to exhaustion (Dunne et al. 2016). At the completion of each exercise bout, the participant transferred immediately to a plinth for ultrasound measurements, alternating continuously between tumour vessel and hepatic artery, for 10 minutes. An overview of the study procedures can be seen in Fig. 1.

The order of intensities performed was randomised using a computer-generated random numbers list. Sealed opaque envelopes, prepared by the PI, containing the randomised exercise sequence were opened in front of the participant by the researcher on the day of testing.

### Outcome measures

Participants completed baseline questionnaires including physical activity behaviour using the International Physical Activity Questionnaire (IPAQ) (Craig et al. 2003), quality of life using the EORTC QLQ-C30 (Aaronson et al. 1993) and fatigue using the FACIT-Fatigue (Webster et al. 2003). Demographic data (age, height, weight, and fat percentage via body impedance analysis (Tanita InnerScan) and clinical information (including cancer diagnosis and medical history) was collected at baseline. During the exercise task and in recovery, heart rate and oxygen saturation (SpO_2_) was measured continuously and recorded every 2-minutes. Blood pressure was measured every 5-minutes. Rating of perceived effort (RPE) (Borg 1982) was measured at the 5th and final minute of the exercise bouts.

Doppler ultrasound was used to measure blood flow in the vessel of the single liver tumour and the hepatic artery, as previously identified immediately after exercise bout completion. Blood flow was unable to be measured during exercise due to movement artifacts. Peak systolic velocity (PSV), end diastolic velocity (EDV), and Resistive Index (RI); calculated as [PSV – EDV]/PSV) were recorded from the Doppler trace, with measures taken alternating between the tumour vessel and the hepatic artery (Supplementary Fig. 1a and b). PSV refers to the velocity of blood flowing into a vessel during systole, EDV refers to the velocity of blood at the end of diastole, and RI refers to the resistance of blood flowing through a vessel (Baik 2010). The sonographer targeted the tumour vessel first (after each exercise segment) to detect maximum effect.

### Statistical analysis

SPSS (IBM SPSS Version 29.0.1.0(171)) was used for the analysis. Wilcoxon signed rank test was used due to the small sample size, with PSV, EDV and RI baseline values compared to values taken in the first 3 min after exercise as the closest estimation of blood flow during exercise. All data are expressed as mean ± standard deviation (SD). *p* < 0.05 was considered significant.

## Results

### Participants

A total of 11 patients were referred for recruitment to the study between December 2022 and October 2024. All patients met the eligibility criteria and eight patients (73%) consented to participate. Reasons for declining participation were all due to pain. Seven of the eight participants had VO_2peak_ > 18 ml/kg/min. One participant had VO_2peak_ ≤18 ml/kg/min (15.14mL/kg/min), however, they completed the three exercise bouts due to a calculation error on the day of testing. No adverse events were recorded throughout the study for any of the participants. Three participants were excluded from analysis due a lack of sonographer confidence in identifying vessels due to the small size and/or difficult to access location of the tumour, resulting in a final analysed sample size of *n* = 5. Demographics and baseline characteristics can be seen in Table 1. The participants in this study had lower fatigue scores than the general metastatic population (Vardy et al. 2014). In terms of quality of life, relative to the average population with metastatic colorectal cancer the participants in this study had similar symptom scores but higher functioning scores (Conroy et al. 2010; Thomsen et al. 2017). Relative to a metastatic population, the participants in this study had a higher level of fitness suggesting greater physical activity levels than the general metastatic population (Yee et al. 2014).

**Table 1:** Demographics and baseline characteristics

	*N* = 5 (%)
Age (years)	46 ± 12.9
Sex	
Female	1 (20)
Male	4 (80)
BMI (kg/m^2^)	22.8 ± 1.7
Muscle (%)	51.7 ± 21.2
Fat (%)	20.1 ± 6.47
Cancer type	
Colorectal	3 (60)
Upper GI	1 (20)
Stomach	1 (20)
Current treatment	
Chemotherapy	5 (100)
Immunotherapy	3 (60)
Estimated VO_2peak_ (mL/kg/min)	28.7 ± 9.9
IPAQ total score (met minutes/week)	3773.7 ± 3187.2
EORTC-QLQ C30	
Global health status	75.0 ± 23.57
Physical	97.33 ± 5.963
Role	76.67 ± 32.48
Emotional	81.67 ± 20.75
Cognitive	90.0 ± 14.91
Social	70.0 ± 3.86
Fatigue	33.33 ± 33.33
Nausea and vomiting	10.00 ± 14.91
Pain	6.67 ± 9.13
Dyspnoea	13.33 ± 18.26
Insomnia	26.67 ± 14.91
Appetite Loss	20.0 ± 18.26
Constipation	26.67 ± 27.89
Diarrhoea	6.67 ± 14.91
Financial difficulties	13.33 ± 18.26
FACIT-Fatigue score	33 ± 13.2

Data presented as mean ± standard deviation (SD)

### Physiological response to exercise

Heart rate, systolic blood pressure and perceived exertion rated on the Borg scale (RPE 6–20) increased as expected in a dose-response pattern with intensity (Table 2). Oxygen saturation remained ≥95% during exercise for all participants.

**Table 2:** Physiological measures during exercise

	Light intensity	Moderate intensity	High intensity
Heart rate (bpm)	103.6 ± 12.7	120.12 ± 10.8	132.0 ± 10.4
Heart rate reserve (%)	27.8 ± 5	44.7 ± 5.8	56.4 ± 3.5
Systolic blood pressure (mmHg)	128.6 ± 12.4	147.2 ± 16.7	165.2 ± 26.0
Diastolic blood pressure (mmHg)	75.6 ± 12.5	75.8 ± 11.2	75.0 ± 11.7
Rate of perceived exertion	8 ± 1.2	10 ± 1.6	13.5 ± 1.5
Power output (watts)	37.4 ± 22.0	78.6 ± 34.7	117.6 ± 53.0
Watts max (%)	18.51 ± 4.42	40.66 ± 5.50	60.42 ± 8.53

Data presented as mean ± standard deviation (SD)

### Blood flow measures

**Fig. 2:**
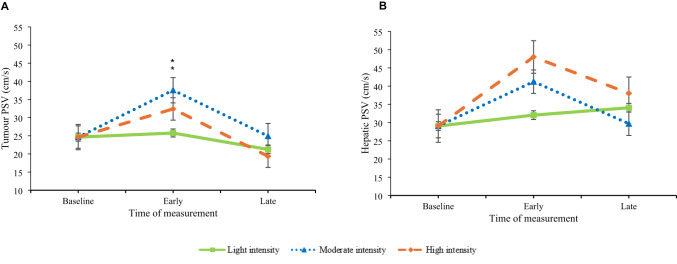
Peak systolic velocity after light, moderate and high intensity exercise. **A** Tumour; **B** Hepatic artery. Early = within first 3-minutes of exercise cessation, late = 10-minutes after exercise cessation. * *p* < 0.05

**Fig. 3:**
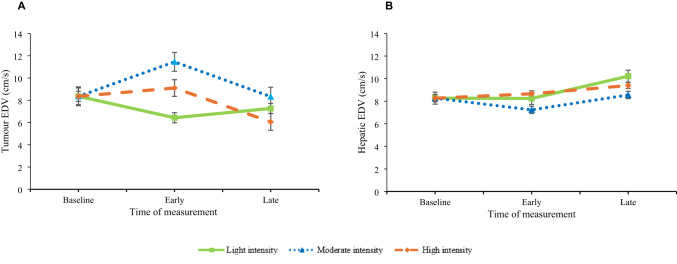
End diastolic velocity after light, moderate and high intensity exercise. **A** Tumour; **B** Hepatic artery. Early = within first 3-minutes of exercise cessation, late = 10-minutes after exercise cessation

**Fig. 4:**
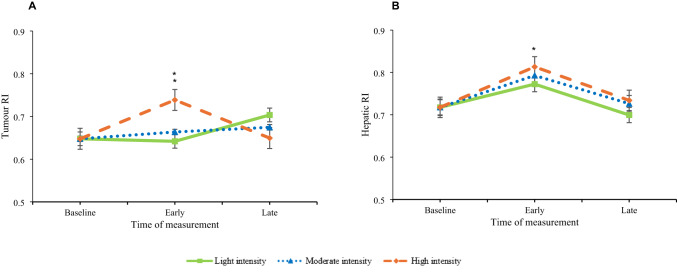
Resistive index after light, moderate and high intensity exercise. **A** Tumour; **B** Hepatic artery. Early = within first 3-minutes of exercise cessation, late = 10-minutes after exercise cessation. * *p* < 0.05

A significant increase of 152% compared to rest was observed in PSV in the tumour vessel immediately after moderate intensity exercise (z = 2.023, *p* = 0.043) and this reduced during late recovery (Figure 2a). There was no significant difference found for PSV in the tumour vessel after the light and high intensity bouts (Fig. 2a), and there was no significant change in EDV found for any exercise intensity in the tumour vessel (Fig. 3a). The RI of the tumour vessel showed a significant increase of 114% (0.74 ± 0.15) after the high intensity bout (z = 2.023, *p* = 0.043) followed by a reduction during late recovery, but no difference after the light or moderate intensity bouts (Fig. 4a).

For the hepatic artery, there was no significance difference between baseline and post exercise in PSV or EDV at any intensities (Figs. 2b and 3b). For the hepatic artery RI measurements, similar to the RI changes in the tumour, there was a significant increase of 113% (0.81 ± 0.15) after the high intensity bout (z = 2.023, *p* = 0.043) followed by a reduction in late recovery, with no differences seen after the light and moderate intensity bouts (Fig. 4b).

## Discussion

This study demonstrates that acute moderate intensity aerobic exercise elicits increased blood flow to metastatic liver tumours in humans, using non-invasive measurement techniques. This result is consistent with the findings of published pre-clinical work. For example, Garcia et al. (2016) measured blood flow in rats with prostate tumours that exercised for 5-minutes at moderate intensity. The authors found ~180% increase in blood flow during exercise compared to rest. Similarly, McCullough et al. (2014) examined rats with prostate tumours that exercised for 5-minutes at moderate intensity and found a 200% increase in blood flow during exercise compared to rest. Our findings likely represent a conservative estimate of increases in blood flow as measures were taken after exercise rather than during exercise, which may explain the smaller magnitude of change observed, and blood flow readings decreased in the later stage of recovery. McCullough and colleagues (2014) also found an increase in the number of patent vessels and coupled with the increase in blood flow and found a ~50% reduction in hypoxia. Although we did not measure hypoxia or number of patent vessels, the similar blood flow findings at the same exercise intensity suggests that changes in patent blood vessels and hypoxia may also occur in a clinical population.

Although there is clinical evidence for the effects of chronic aerobic exercise on tumour blood flow (Florez Bedoya et al. 2019; Jones et al. 2013; Schumacher et al. 2025), there is only one other clinical study to our knowledge that investigated the effects of acute aerobic exercise on the tumour microenvironment. Djurhuus et al. (2022) investigated whether a single bout of high intensity interval exercise caused tumour microenvironment remodelling in men with prostate cancer. Participants cycled for 4 × 1-minute high intensity bouts (100% of peak power output) one day prior to a radical prostatectomy. Tumour microvessel density was assessed using tumour tissue sections taken during the prostatectomy but tumour blood flow was not measured. The authors found that there was no change in hypoxia or microvessel density compared to no exercise the day prior to surgery. It is possible that despite a lack of change in hypoxia and vessel density, acute blood flow changes occurred but were not sustained.

In the current study, we found that low and high intensity exercise did not elicit significant blood flow changes to the tumour. Given the lack of vasoconstrictive response in tumours, we expected to see a linear increase in tumour blood flow in parallel to the increase in cardiac output with increasing exercise intensities. In the low intensity exercise bout, it is possible that the increase in cardiac output did not sufficiently increase blood flow to the host tissue (liver) and therefore did not change tumour blood flow. In line with pre-clinical work, Suzuki et al. (1981) demonstrated that liver blood flow increased with pharmacologically induced hypertension up to 145 mmHg, but then declined. Our results are consistent with this finding, with systolic blood pressure during moderate exercise ~147mmHg, which may have been optimal for enhancing tumour blood flow. In contrast, during high intensity exercise, where systolic blood pressure was ~162mmHg, we found no difference from baseline. Although care should be taken when translating pressure differences between humans and animals, the greater blood pressures elicited during high intensity exercise may have induced a vasoconstrictive response in the hepatic artery that shunted blood away from the host tissue (liver) (Trefts 2022) and resulted in the lower PSV in the tumour.

We observed a significant increase in tumour and hepatic RI with high intensity exercise with no significant change in PSV or EDV. Given that hepatic RI increased likely due to vasoconstriction (Trefts 2022), it is possible that there was also a vasoconstrictive response in the tumour elicited by the higher intensity. It may also relate to the heterogenous tumour blood vessel distribution and dysfunction interacting with changed cardiovascular variables. This finding may reflect changes in vessel function at higher pressures, but it should also be acknowledged that technical difficulty in obtaining accurate readings due to the movement artefacts of heavy respiration post-exercise could also contribute to these findings. Further examination of vessel responses to higher blood pressures such as those elicited by higher intensity exercise may provide further elucidation.

Given there are few clinical studies, the implications of our results are significant. This study has demonstrated that moderate intensity exercise acutely increased blood flow to metastatic liver tumours by 152% immediately post exercise. This result is likely a conservative estimate of blood flow changes during exercise such that blood flow would likely be increased to the greatest extent during exercise performance. If generalisable to other solid tumours, this effect might be harnessed to enhance drug delivery if exercise is performed during treatment. Exercise performed simultaneous to chemotherapy infusion, termed intra-infusion exercise, has been proposed as a novel adjuvant to treatment using this mechanism. Pilot studies have demonstrated that low intensity intra-infusion exercise is safe and feasible (Kerrigan et al. 2022, McLaughlin et al. 2019, Thomas et al. 2019). Combined with our findings, these studies support the need for future work to examine the clinical effect of intra-infusion exercise to determine if increased blood flow elicited by exercise subsequently affects treatment efficacy as hypothesised through increasing drug delivery to target tumours.

It is also important to consider the potential contribution of chronic exercise-induced adaptations. Repeated bouts of aerobic exercise (exercise training) can induce adaptions to the tumour vasculature such as increased blood vessel density, improved organisation and improved vessel function (Seet-Lee et al. 2022). These vasculature adaptations have been suggested to be stimulated by the cumulative effects of each acute bout. We provide initial evidence for the acute effect of exercise on tumour blood flow which may be used to further understand the chronic adaptations. Similarly, it is important to recognise that chronic exercise-induced adaptations may also augment acute blood flow delivery by increasing the number of normalised blood vessels (Seet-Lee et al. 2022). We saw that our cohort had higher mean estimated VO_2peak_ and IPAQ score relative to patients with metastatic liver cancer (Dunne et al. 2016) suggesting that the participants performed repeated bouts of aerobic exercise that may have induced tumour microenvironment adaptations. Due to the small sample size, we cannot assess the association between physical fitness and tumour blood flow, but we note that the participant with the highest predicted VO_2peak_ had the greatest PSV response after moderate intensity exercise. Future research with larger sample sizes should consider tumour vascular adaptations from chronic exercise that may influence acute blood flow delivery effects.

This study has several limitations. It included a very small sample size reflecting the difficulty in recruiting people with advanced metastatic cancer, well enough for oncologist referral to an exercise trial, to complete a single visit exercise trial with no likely individual benefit. Furthermore, it was not possible to measure blood flow with Doppler ultrasound whilst exercising due to movement artefact. Although we were able to obtain tumour vessel measures mostly within one minute post exercise, the sudden cessation of exercise along with the slight delay in measurement and the change in position from sitting to supine likely meant blood flow was lower than would have been observed during exercise.

## Conclusion

In summary, this study provides initial clinical evidence that acute moderate intensity aerobic exercise increases blood flow to liver tumours. While replication in larger samples is essential, this study is a step toward the potential integration of aerobic exercise prescription into standard cancer treatment as a potential adjuvant strategy to improve chemotherapy delivery and efficacy.

## Supplementary Information

Below is the link to the electronic supplementary material.

Supplementary Material 1

## Data Availability

The datasets generated and/or analysed during the current study are available from the corresponding author on reasonable request.
