# Supplementary material for: The effect of acute aerobic exercise intensity on indices of blood flow to metastatic liver tumours using non-invasive imaging: a preliminary study
Source: Eur J Appl Physiol. 2026 Mar 11;126(7):3787–94. doi: 10.1007/s00421-026-06139-3 (PMC13380594; doi:10.1007/s00421-026-06139-3)
Supplement: Supplementary file 1 — Supplementary Material 1 [file 421_2026_6139_MOESM1_ESM.docx]

**Supplementary**

|  | Tumour | | | Hepatic | | |
| --- | --- | --- | --- | --- | --- | --- |
|  | Light | Moderate | High | Light | Moderate | High |
| Baseline | 24.63±5.66 | 24.63±5.66 | 24.63±5.66 | 29.05±6.69 | 29.05±6.69 | 29.05±6.69 |
| Early | 25.76±7.63 | 37.56±5.91^+^ | 32.4±12.52 | 32.04±10.11 | 41.22±15.71 | 48.0±17.03 |
| Late | 21.24±6.67 | 24.92±4.56 | 19.36±5.08 | 34.06±7.70 | 29.7±6.25 | 38.02±13.54 |

Table 1. Mean PSV data in tumour and hepatic artery after light, moderate and high intensity exercise. N=5. Data is presented as mean±SD. ^+^ p<0.05

|  | Tumour | | | Hepatic | | |
| --- | --- | --- | --- | --- | --- | --- |
|  | Light | Moderate | High | Light | Moderate | High |
| Baseline | 8.35±3.72 | 8.35±3.72 | 8.35±3.72 | 8.27±4.48 | 8.27±4.48 | 8.27±4.48 |
| Early | 6.43±1.15 | 11.45±3.80 | 9.1±7.76 | 8.24±3.12 | 7.23±0.96 | 8.66±2.54 |
| Late | 7.26±1.45 | 8.33±3.84 | 6.05±3.56 | 10.21±4.23 | 8.55±3.92 | 9.39±2.88 |

Table 2. Mean EDV data in tumour and hepatic artery after light, moderate and high intensity exercise. N=5. Data is presented as mean±SD. ^+^ p<0.05

|  | Tumour | | | Hepatic | | |
| --- | --- | --- | --- | --- | --- | --- |
|  | Light | Moderate | High | Light | Moderate | High |
| Baseline | 0.65±0.14 | 0.65±0.14 | 0.65±0.14 | 0.72±0.14 | 0.72±0.14 | 0.72±0.14 |
| Early | 0.64±0.56 | 0.66±0.14 | 0.74±0.15^+^ | 0.77±0.09 | 0.79±0.11 | 0.81±0.15^+^ |
| Late | 0.70±0.18 | 0.67±0.13 | 0.65±0.21 | 0.70±0.12 | 0.73±0.12 | 0.73±0.16 |

Table 3. Mean RI data in tumour and hepatic artery after light, moderate and high intensity exercise. N=5. Data is presented as mean±SD. ^+^ p<0.05

|  | Tumour | | Hepatic | |
| --- | --- | --- | --- | --- |
|  | Pre-YMCA | Post-YMCA | Pre-YMCA | Post-YMCA |
| PSV | 28.5±13.48 | 24.63±5.66 | 38.0±18.19 | 29.05±6.69 |
| EDV | 9.11±6.85 | 8.35±3.72 | 10.27±3.49 | 8.27±4.48 |
| RI | 0.68±0.14 | 0.65±0.14 | 0.71±0.10 | 0.72±0.14 |

Table 4. Pre-YCMA and post-YMCA blood velocities at rest. N=5. Data is presented as mean±SD.

|  | Low intensity | Moderate intensity | High intensity |
| --- | --- | --- | --- |
| Order of intensity |  |  |  |
| 1^st^  PSV (cm/s)  EDV (cm/s)  RI  %HRR (bpm)  %watts max (watts) | N=2  33.7±0.42  6.34±1.63  0.63±0.1  27.48±1.27  20.0±0 | N=2  31.6±1.84  10.47±0.75  0.66±0.01  44.28±4.06  40.70±1.37 | N=1  38.6±0  7.49±0  0.84±0  58.23±0  69.44±0 |
| 2^nd^  PSV (cm/s)  EDV (cm/s)  RI  %HRR (bpm)  %watts max (watts) | N=2  19.9±4.53  6.06±1.22  0.65±0.03  27.31±2.98  18.78±7.82 | N=1  39.8±0  6.92±0  0.83±0  50.0±0  43.0±0 | N=2  34.9±18.24  14.71±11.02  0.62±0.09  57.14±2.04  58.33±11.79 |
| 3^rd^  PSV (cm/s)  EDV (cm/s)  RI  %HRR (bpm)  %watts max (watts) | N=1  21.6±0  7.5±0  0.66±0  27.27±0  15.0±0 | N=2  42.4±3.68  14.7±3.68  0.58±0.19  46.66±3.33  39.43±10.52 | N=2  26.8±13.44  4.31±2.82  0.81±0.18  63.06±5.10  57.99±7.09 |

Table 5. Randomisation order on tumour PSV early, tumour EDV early, tumour RI early, percentage heart rate maximum during exercise and percentage watts maximum.


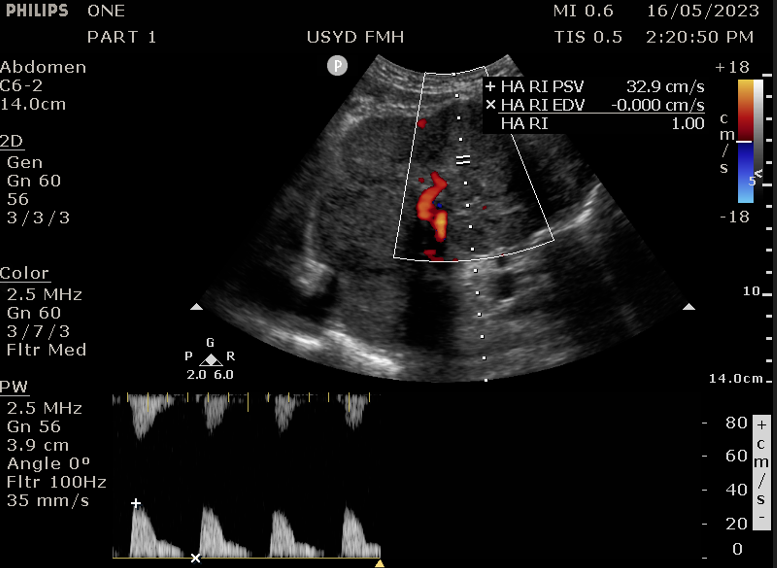
**A**  **B**


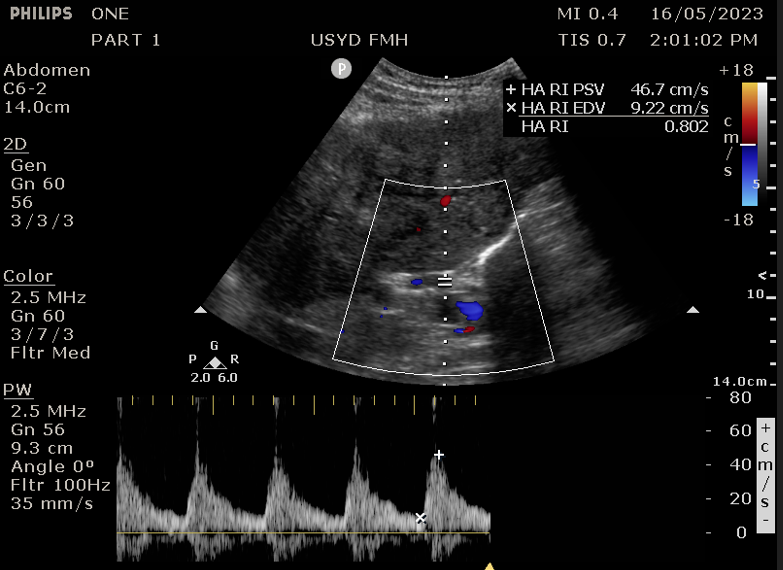


Fig 1. Doppler trace of participants immediately after exercise. A Hepatic artery; B Liver tumour vessel
